# Challenges in the diagnosis and treatment of candidemia due to multidrug-resistant *Candida auris*

**DOI:** 10.3389/ffunb.2023.1061150

**Published:** 2023-02-02

**Authors:** Daniele Roberto Giacobbe, Malgorzata Mikulska, Antonio Vena, Vincenzo Di Pilato, Laura Magnasco, Anna Marchese, Matteo Bassetti

**Affiliations:** ^1^ Department of Health Sciences (DISSAL), University of Genoa, Genoa, Italy; ^2^ Clinica Malattie Infettive, IRCCS Ospedale Policlinico San Martino, Genoa, Italy; ^3^ Department of Surgical Sciences and Integrated Diagnostics (DISC), University of Genoa, Genoa, Italy; ^4^ Unità di Microbiologia, IRCCS Ospedale Policlinico San Martino, Genoa, Italy

**Keywords:** Candida auris, candidemia, diagnosis, treatment, MDR

## Introduction

*Candida* infections have been increasingly recognized as a serious concern for critically ill patients and/or immunocompromised individuals with serious underlying diseases (Pappas et al., 2018). Although *C. albicans* accounts for most of invasive infections, non-albicans species are becoming more frequent (Giacobbe et al., 2020). Among these, *C. auris* garnered major attention due to its variable antifungal resistance profiles and ability to disseminate within the health-care setting (Jeffery-Smith et al., 2018; Giacobbe et al., 2021).

Although many sites of infection have been reported (Choi et al., 2017; Heath et al., 2019; Khatamzas et al., 2019; Roberts et al., 2019; Shenoy et al., 2019; Supreeth et al., 2020; Shaukat et al., 2021; Mirhendi et al., 2022), candidemia remains the most common invasive infection caused by *C. auris*. Supported by the available evidence, in this article we share our view on the current challenges in the diagnosis and treatment of candidemia by *C. auris*, in particular when caused by multidrug-resistant (MDR) isolates.

## Identification of *C. auris* and assessment of resistance patterns

The emergence of *C. auris* posed important challenges regarding laboratory detection and infection control measures (ECDC, 2018; Keighley et al., 2021). Indeed, the common misidentification by diagnostic platforms available in clinical microbiology and public health laboratories, a poor understanding of resistance to antifungal drugs and disinfectants, together with the ability to persistently colonize abiotic and biotic surfaces, were deemed among major factors contributing to the rapid spread of *C. auris* within the healthcare system (Desoubeaux et al., 2022).

Standard culture-based approaches do not permit differentiation of *C. auris* from other common *Candida* spp. as it lacks a distinctive traits, although newly formulated chromogenic media gave promising results for its rapid presumptively identification based on colony color and appearance (Borman et al., 2021). Furthermore, the tolerance to growth temperature up to 42°C (unlike many other *Candida* species), as well as the inability to make hyphae or pseudohyphae on Corn Meal agar plates (as generally observed for *C. guilliermondii, C. lusitaniae*, and *C. parapsilosis*), could be of some help to differentiate between *C. auris* and related species, although none of the former methods is ideal for an accurate species confirmation. On the other hand, the use of salt/dulcitol selective media (i.e., characterized by a high salinity - 10% NaCl - and by a carbon source based on dulcitol rather than glucose) could represent a useful strategy to selectively screen for *C. auris* in non-sterile body sites (CDC, 2022).

The use of biochemistry-based methods similarly poses major problem in an accurate identification of *C. auris*, since its biochemical assimilation profile is very similar to that of other closely related species, most commonly belonging to the *C. haemulonii* complex (but also *C. famata*, *C. sake*, *Rhodotorula glutinis*, *R. mucilaginosa*, and *Saccharomyces* spp.), leading to a great deal of misidentification (Jeffery-Smith et al., 2018; Lockhart et al., 2022). Some incremental improvements have been achieved by updating databases of most common biochemical platforms (i.e., Vitek 2, MicroScan Walkaway, BD Phoenix), but a suboptimal *Candida* spp. identification has been reported and discrepancies can arise. To overcome these issues, multiple guidance algorithms have been proposed to identify *C. auris* based on phenotypic laboratory methods and initial species identification (CDC, 2019).

At present, a reliable recognition of *C. auris* can be definitively achieved by mass spectrometry MALDI-TOF (i.e., bioMérieux Vitek MS, Bruker Biotyper 2.0 Microflex LT), provided that an up-to-date spectra database is used (Keighley et al., 2021). Considering the recent global emergence of this fungal pathogen, diagnostic microbiology laboratories should check with the manufacturer concerning the presence of *C. auris* in the database of their identification platforms and, if not available, referral of non-albicans *Candida* spp. invasive isolates to a reference laboratory is advisable (ECDC, 2018). To help fill this gap, molecular PCR-based assays relying on amplification and sequencing of D1/D2, RPB1/RPB2 and ITS loci have been increasingly adopted as complementary identification tests (Jeffery-Smith et al., 2018), whereas the superior discriminative power of WGS proved extremely useful to resolve new introduction vs. local transmission events in outbreak settings (Lockhart et al., 2017; Di Pilato et al., 2021; Salah et al., 2021). Noteworthy, among the currently marketed syndromic tests employed for the rapid diagnosis of bloodstream infections, which are the most powerful molecular tools that may assist in a timely manner diagnosis of infections, very few tests (e.g. the GenMark ePlex Blood Culture Identification Fungal Pathogen Panel and BioFire FilmArray BCID2 panels) include *C. auris* within the target organisms (Dumkow et al., 2021).

Although misidentification is potentially problematic, the reduced susceptibility to some antifungal agents (Keighley et al., 2021), as well as the rapid emergence of MDR isolates (i.e., exhibiting resistance to at least two antifungal classes) upon antifungal treatment (Jacobs et al., 2022; Rybak et al., 2022), are among the major causes of concern from *C. auris* infections.

Further concerns are related to antifungal susceptibility testing (AFST), which is complicated by the lack of *C. auris*-specific interpretative breakpoints, yet to be reported by both the Clinical Laboratory Standards Institute (CLSI) and the European Committee for Antimicrobial Susceptibility Testing (EUCAST). Strikingly, there are not likely to be established breakpoints in the near future either, since clinical trials of currently available antifungal agents against *C. auris* are missing. Nevertheless, based on available pharmacokinetic/pharmacodynamic data, tentative interpretative criteria have been proposed by the CDC (i.e., to be used with the reference broth microdilution method endorsed by CLSI) (CDC, 2020).

According to the CDC breakpoints, a high frequency of fluconazole- and amphotericin B-resistant isolates was observed (surpassing 90% and 30%, respectively), representing a specific phenotypic signature of *C. auris* since its emergence in 2009. On the other hand, a reduced susceptibility to other triazole antifungals (i.e., voriconazole, itraconazole, and isavuconazole) and echinocandins was observed less frequently (Garcia-Bustos et al., 2021; Sanyaolu et al., 2022).

An additional challenge diagnostic laboratories should be aware of involves determination of echinocandins MICs, mostly caspofungin, for which AFST may be impacted by the paradoxical growth effect (i.e., also known as Eagle effect), a phenomenon that could lead to major errors in predicting echinocandin resistance in a susceptible isolate (Briano et al., 2022; Kordalewska and Perlin, 2022). As such, confirmation of AFST results by a reference laboratory is advisable.

## Treatment in real-life experiences

Previous studies report a prevalence of candidemia among colonized patients of around 17% (Schelenz et al., 2016; Garcia-Bustos et al., 2020), with an estimated cumulative incidence up to over 25% with increasing length of stay in critically ill subjects (Briano et al., 2022). Despite many reports of *C. auris* candidemia, coherent data on efficacy of active antifungal therapy are scanty, and hampered by small sample sizes, heterogeneity of treatment, and lack of adjustment for confounding factors (see **Table 1**). For example, many studies reported the use of antifungals with no *in vitro* activity. In two large Spanish studies, in which all strains were susceptible to echinocandins and echinocandins were included in the treatment regimen in all 41 and 47 patients, 30-day mortality was, respectively, 41% and 23% (Ruiz-Gaitan et al., 2018; Mulet Bayona et al., 2020).

**Table 1:** Summary of current available evidence on the treatment of *C. auris* candidemia.

Reference Setting	Number of patients included	Treatment administered and treatment duration (when available)	Outcome reported	Resistance to antifungal classes of isolated strains
(Prayag et al., 2022) Western India	34	Not reported	Mortality: 32%	Fluconazole: 100% Amphotericin B: 32% Echinocandins: 6% (n=2) for caspofungin and 3% (n=1) for micafungin
(Allaw et al., 2022) Lebanon	8	Echinocandin	Mortality: 75%	Fluconazole: not reported Amphotericin B: not reported Echinocandins: 0%
(Reque et al., 2022) Spain	1	Amphotericin B 100 mg/day (21 days) + anidulafungin 100 mg/day (14 days)	Survived	Fluconazole: not reported Amphotericin B: not reported Echinocandins: not reported
(Tsai et al., 2022) Taiwan	1	Anidulafungin 200/100 mg/day (15 days)	Survived	Fluconazole: 0% Amphotericin B: 0% Echinocandins: 0%
(Pandya et al., 2021) 10 centres from 5 countries	41 (additional 7 without candidemia) Treatment data available for 47 episodes of infection	83% echinocandin 11% fluconazole 2% voriconazole 2% L-AmB 2% L-AmB + caspofungin N=7 not treated Source removal 74%	30-day mortality: 37% Lower mortality associated with treatment (OR 0.27) and source removal (OR 0.74) Microbiological clearance in 60% of treated	Data on resistance available for 54 isolates Fluconazole: 78% Amphotericin B: 57% Echinocandins: 0% micafungin, 5% anidulafungin, 16% caspofungin
(Moin et al., 2021) Pakistan	4	100% amphotericin B 75% fluconazole 75% voriconazole 25% caspofungin	Mortality: 67%	Fluconazole: 100% Amphotericin B: 0% Echinocandins: 0%
(Hanson et al., 2021) US	8 Data available for 15 isolates in total	6/8 micafungin (subsequent amphotericin B in 1 case) 2/8 data not available	Mortality: 63%	Fluconazole: 100% Amphotericin B: 0% Echinocandins: 7% (n=1, resistant to all echinocandins)
(Berrio et al., 2021) Colombia	34 paediatric patients, 68% <1 year of age	47% amphotericin B deoxycholate 29% azoles 21% caspofungin	In-hospital mortality: 41%	Data available for 13/34 strains Fluconazole: 15% Amphotericin B: 54% Echinocandins: 8% (n=1, resistant to anidulafungin and AmB but susceptible to micafungin and caspofungin)
(Ayala-Gaytan et al., 2021) Mexico	1	Caspofungin 70/50 mg/day + L-AmB 3mg/kg, 18 days	Survived	Fluconazole: 100% Amphotericin B: 0% Echinocandins: 0%
(Mohsin et al., 2020) Oman	23	All echinocandins, some patients received later combination with amphotericin B, mean duration of treatment 13 days	Mortality: 39%	Fluconazole: 100% Amphotericin B: 22% Echinocandins: 0%
(Mulet Bayona et al., 2020) Spain	47 (only first episodes included)	47% echinocandin monotherapy 26% echinocandin + amphotericin B 21% echinocandin + isavuconazole Median duration of treatment 21 days	30-day mortality: 23% Recurrence of candidemia: 15% persistent candidemia: 12.8% (n=6); Endophthalmitis 4.3% (n=2)	Fluconazole: 100% Amphotericin B: 0% Echinocandins: 0%* *1 case of emergence of resistance to echinocandin in a patient from whom a susceptible strain was isolated two months earlier from CVC-related candidemia treated with anidulafungin for 28 days and antifungal lock of the catheter with anidulafungin until the catheter was replaced.
(Barantsevich et al., 2020) Russia	38	13% caspofungin 70/50 mg/day 8% micafungin 150 mg/day 66% fluconazole 3% voriconazole 3% amphotericin B 12.5 mg, 10 days	Mortality: 55%	Fluconazole: 97% Amphotericin B: 76% Echinocandins: 0%
(Chowdhary et al., 2020) India	15	60% micafungin 13% amphotericin B 8% micafungin + amphotericin B	Mortality: 53%	Fluconazole: 100% Amphotericin B: 40% Echinocandins: 0% *data available only for 10/15; 70% MDR strains.
(Chandramati et al., 2020) India	17 neonates	18% voriconazole monotherapy 12% fluconazole monotherapy 6% fluconazole + voriconazole 12% voriconazole + micafungin 29% amphotericin B + micafungin 24% amphotericin B + voriconazole	Mortality: 41% 17% in those treated with a combination of micafungin and amphotericin B	Fluconazole: 100% Amphotericin B: not reported Echinocandins: 0%
(Bajpai et al., 2020) India	5	60% caspofungin 40% voriconazole	Mortality: 40%	Fluconazole: 100% Voriconazole: 40% Amphotericin B: 50% Echinocandins 40% (1 strain resistant to both caspofungin and micafungin; other 2 strains resistant to only one of two echinocandins)
(Shastri et al., 2020) India	42	60% caspofungin 10% micafungin 2% anidulafungin 14% amphotericin B deoxycholate 10% L-AmB 5% no treatment	30-day mortality: 67%; attributable mortality: 31%	Fluconazole: 100% Amphotericin B: 71% Echinocandins: 3% (n=1/33, resistant to caspofungin, susceptible to micafungin)
(Ninan et al., 2020) South India	11	45% fluconazole 27% amphotericin B 9% caspofungin 18% no treatment	Mortality: 18%	Fluconazole: 91% Amphotericin B: 0% Echinocandins: 0%
(Taori et al., 2019) UK	8 (5 CLA-BSI) in 34 patients who acquired *C. auris* colonization	88% echinocandin monotherapy 13% amphotericin B monotherapy 13% amphotericin B following or concomitant to echinocandin 26% voriconazole following or concomitant to echinocandin	30-day mortality: 25%	Data available for 54 strains, including the colonising ones, but interpretation provided based on CLSI criteria *for C. albicans*. Fluconazole: 100% Amphotericin B: 30% Echinocandins: 12% (5/41) for anidulafungin, but only 1 strain with MIC ≥1 40% (4/10) for caspofungin, but only 1 strain with MIC >32, all others <2
(Park et al., 2019) USA	9	Micafungin 100 mg/day in all, increased to 150 mg/day in 3 cases, mean 22 days In 2 cases with reported failure, L-AmB 4-5 mg/Kg/day was added for 19 days	In-hospital mortality: 22%	Fluconazole: 100% Amphotericin B: 37.2% Echinocandins: 0% Data available for 8 strains
(Sayeed et al., 2019) Pakistan	38 Data available for 65 episodes of invasive infection	Amphotericin B deoxycholate 0.75 mg/kg as first line, alternative voriconazole in case of renal impairment (LD 6 mg/kg q12h, MD 4 mg/kg q12h).	Mortality: 77% (only patients with candidemia)	Data available for 63 strains: Fluconazole: 100% Amphotericin B: 7.9% Echinocandins: 0%
(Armstrong et al., 2019) Colombia	40	40% fluconazole 33% caspofungin 20% amphotericin B 3% voriconazole 48% combination treatment 5% no treatment	30-day mortality: 43%	Fluconazole: 18% Amphotericin B: 29% Echinocandins: 0% Data available for 34 strains
(Alatoom et al., 2018) United Arab Emirates	1	Caspofungin then amphotericin B	Died	Fluconazole: not reported Amphotericin B: 0% Echinocandins: 0%
(Ruiz-Gaitan et al., 2018) Spain	41	Echinocandin Combination therapy with echinocandin + L-AmB in 40%	30-day mortality: 41%	Fluconazole: 100% Amphotericin B: 0% Echinocandins: 0%
(Khan et al., 2018) Kuwait	11 Data available for 18 cases of invasive infection	33% caspofungin 11% fluconazole 6% L-AmB 6% voriconazole + caspofungin 11% voriconazole 6% L-AmB then fluconazole + voriconazole 6% caspofungin then L-AmB	Mortality: 64% (only patients with candidemia)	Fluconazole: 100% Amphotericin B: 23.5% Echinocandins: 0%
(Chen et al., 2018) China	2	Fluconazole then itraconazole	Survived	Fluconazole: 100% Amphotericin B: 0% Echinocandins: 0%
(Ben-Ami et al., 2017) Israel	5 Data available for 9 patients, 3 with *C. haemulonii* infection	80% fluconazole and/or anidulafungin 20% fluconazole then voriconazole 20% only removal of CVC	Mortality: 40%	Fluconazole: 60% Amphotericin B: 0% Echinocandins: 0%
(Mohsin et al., 2017) Oman	2	Anidulafungin Caspofungin	In-hospital mortality: 50%	Fluconazole: 100% Amphotericin B: 0% Echinocandins: 0%
(Calvo et al., 2016) Venezuela	18	Variable (fluconazole, voriconazole, amphotericin B, caspofungin, anidulafungin) * * Unclear whether sequential or combination therapy	30-day mortality: 28%	Fluconazole: 100% Amphotericin B: 0% Echinocandins: 0%
(Lee et al., 2011) South Korea	3	Fluconazole then amphotericin B	Mortality: 67%	Fluconazole: not clearly reported, MIC 2 to 128 mg/L Amphotericin B: 0% Echinocandins: 0%
(Simon et al., 2022) USA	83	Micafungin (89%) Caspofungin (11%)	30-day mortality: 30%	Micafungin: 0% (0/62) Anidulafungin: 0% (0/62) Caspofungin: 3.2 (2/62) Amphotericin B: 86% (38/44, YeastOne) and 67% (32/48, Etest) Fluconazole: 100%

CVC, central venous catheter; MDR, multidrug-resistant; CLA-BSI, central line associated-blood stream infection; MIC, minimum inhibitor concentration; LD, loading dose; MD, maintenance dose.

A necessary premise is that the prompt identification of *C. auris* colonization with the implementation of screening protocols in high-risk areas, and the subsequent put in place of adequate infection control measures to prevent cross-transmission of this pathogen among health-care facilities (such as contact precautions, cohorting or isolation of colonized patients) remain pivotal in the prevention of invasive *C. auris* infections, and thus of the consequent impact of *C. auris* on patients’ outcome. However, when *C. auris* candidemia develops, antifungal therapy should be promptly initiated. To date, echinocandins are the mainstay of *C. auris* candidemia treatment. Indeed, while resistance to fluconazole and polyenes may reach over 90% and 30%, respectively, resistance to echinocandins is around 5-7%, although important differences exist across studies (see **Table 1**) (Garcia-Bustos et al., 2021; Sanyaolu et al., 2022). High rates of MDR (intended as resistance to at least two classes of antifungals and reaching up to 25-50%) do jeopardize the possibility of second line treatments in patients who either fail or develop complications to first-line treatment with echinocandins (Garcia-Bustos et al., 2021; Sanyaolu et al., 2022; Vinayagamoorthy et al., 2022). In addition, biofilm production contributes significantly to hamper treatment of *C. auris* infections, through contributing to resistance to antifungals by efflux pumps or inhibition of drug diffusion, and by increasing the probability of persistent infection in case of inadequate source control (Sanyaolu et al., 2022).

There are worrisome reports of emerging resistance to echinocandins after a first treatment course with these antifungals, particularly in case of catheter-related infections (Biagi et al., 2019; Al-Obaid et al., 2022; Briano et al., 2022; Mulet-Bayona et al., 2022). These reports highlight how adequate source control is an essential intervention to improve treatment success and possibly prevent induction or selection of resistance (Biagi et al., 2019; Mulet-Bayona et al., 2022). Once again, however, a clear report of the number of patients who needed to switch to second line treatments is missing, although much needed to better understand the impact of antifungal resistance in clinical practice. For example, combinations of antifungals have been tested mostly in *in vitro*. Synergism was noted for echinocandins and azoles combinations, in particular for anidulafungin or micafungin and isavuconazole (Caballero et al., 2021), anidulafungin and isavuconazole (but not anidulafungin and voriconazole) (Caballero et al., 2021), and micafungin and voriconazole, while no synergy was observed for voriconazole and caspofungin (Fakhim et al., 2017). *In vitro* evidence of non-fungicidal activity of echinocandins monotherapy was also reported, supporting the interest in drug combinations (Caballero et al., 2021). No antagonism, but also no synergy (except for one strain), was reported for flucytosine-based combinations, including amphotericin B, micafungin and voriconazole (Bidaud et al., 2019; O'Brien et al., 2020). Synergy between amphotericin B and micafungin was demonstrated in 8 among 10 tested strains (Jaggavarapu et al., 2020). Of note, in addition to known limitations of *in vitro* synergy models, activity of drug combinations may be not only species-specific but also strain-specific (Caballero et al., 2021).

## Novel drugs in clinical development

Some novel antifungal agents are in advanced phases of clinical development that, for the first time in years, could improve/expand spectrum of activity, route of administration, drug-drug interactions, tolerability of currently available antifungals (Rauseo et al., 2020; Hoenigl et al., 2021; Jacobs et al., 2021).

Rezafungin is a second generation echinocandin, administered intravenously once weekly thanks to its prolonged half-life (Sandison et al., 2017). Its activity against *C. auris* was investigated in two invasive candidiasis models of immunocompromised mice, in which rezafungin exhibited potent *in vivo* activity (Lepak et al., 2018) and performed better than amphotericin B or micafungin in terms of kidney tissue penetration (Hager et al., 2018b). A phase II randomized double-blind study (STRIVE), including 207 patients (none with *C. auris* infection), safety and efficacy of rezafungin was similar to caspofungin for treating candidemia and/or invasive candidiasis (Thompson et al., 2021). Very recently, the results of a noninferiority, double-blind study (RESTORE), comparing rezafungin vs. caspofungin for treating invasive candidiasis and/or candidemia have been released (Thompson et al., 2022). The 14-day overall cure (clinical cure, microbiological cure and radiological cure) in the modified ITT population was 60.6% (57/94) and 59.1% (55/93) in caspofungin-treated and rezafungin-treated patients, respectively (95% CI for difference -14.9 to 12.7).

Fosmanogepix (APX001), the prodrug of manogepix (APX001A, E1211), inhibits the inositol acyltransferase enzyme (Gtw1) involved in the trafficking and anchoring of mannoproteins on the fungal wall (Shaw and Ibrahim, 2020). It displays potent fungistatic activity against most pathogenic *Candida* spp., including *C. auris* (MIC_90_ ≤0.12 mg/ml), although not against *C. krusei* and *C. kefyr* (Miyazaki et al., 2011). The drug is available in both oral and intravenous formulations (Shaw and Ibrahim, 2020), distributes well to many difficult-to-treat body sites and shows a favorable drug-drug interaction profile. In a murine model of disseminated *C. auris* infection, survival was 80-100% and 50% in fosmanogepix-treated and anidulafungin-treated animals, respectively (Hager et al., 2018a). In a phase II, single arm study, including 21 non-neutropenic patients with candidemia treated with fosmanogepix, success rate was 80% (16/20) (Pappas et al., 2020). Among nine critically ill subjects with *C. auris* candidemia, treatment success at end of fosmanogepix treatment and 30-day survival were both 89% (Kullberg et al., 2021).

Ibrexafungerp (SCY-078 or MK-3118) is a 1,3-beta-D-glucan synthase inhibitor with fungicidal activity against *Candida* spp., including *C. auris* (Ghannoum et al., 2018). Compared to echinocandins, ibrexafungerp binds a different site of the same target, so cross-resistance is limited (Jimenez-Ortigosa et al., 2017). Differently from echinocandins, ibrexafungerp is administered orally. An intravenous formulation has also completed phase 1 of clinical development (SCYNEXIS, 2021). In preclinical models, ibrexafungerp was shown to improve survival of neutropenic mice with *C. auris* invasive candidiasis (Wiederhold et al., 2021). In recently published phase II study, oral ibrexafungerp following initial echinocandin therapy was compared to standard of care (step-down to fluconazole) in non-neutropenic patients with invasive candidiasis. Favorable clinical response was 71%, 86%, and 71% in patients receiving ibrexafungerp 500 mg, ibrexafungerp 750 mg, and fluconazole (Spec et al., 2019). One phase III open-label study (CARES, NCT03363841) specifically evaluating the safety and efficacy of ibrexafungerp for *C. auris* infection is currently ongoing. In the first ten patients enrolled, complete response was 80% (Juneja et al., 2021). Other studies assessing the safety and efficacy of oral ibrexafungerp for invasive candidiasis are currently ongoing (FURI, NCT03059992 and MARIO, NCT 05178862).

## Conclusion

Despite various factors (e.g., concomitant infectious and non-infectious diseases) very likely contribute to the prognosis of patients with *C. auris* candidemia, especially if critically ill, proper diagnosis and antifungal treatment remain pivotal to improve cure rates. While some crucial improvements in the diagnosis of *C. auris* candidemia have been observed in the last decade, there is still uncertainty about the best approach for treating MDR *C. auris* candidemia when echinocandins are unavailable due to resistance or other reasons, leading to wide heterogeneity of approaches across studies and likely relying on the current lack of high certainty data. The availability of novel antifungal agents could provide both additional (possibly first line) options and additional clinical data for both reducing heterogeneity and improving treatment of MDR *C. auris* candidemia in daily practice.

## Author contributions

Conceptualization, DG, MM, AM, and MB. writing—original draft preparation, DG, LM, VDP, MM, and AV. writing—review and editing, DG, LM, VDP, MM, AV, AM, and MB. supervision, DG, MM, AM, and MB. All authors contributed to the article and approved the submitted version.
